# Influence of Specific Treatment Parameters on Nontarget and Out-of-Field Doses in a Phantom Model of Prostate SBRT with CyberKnife and TrueBeam

**DOI:** 10.3390/life12050628

**Published:** 2022-04-23

**Authors:** Marta Kruszyna-Mochalska, Agnieszka Skrobala, Piotr Romanski, Adam Ryczkowski, Wiktoria Suchorska, Katarzyna Kulcenty, Igor Piotrowski, Dorota Borowicz, Kinga Graczyk, Natalia Matuszak, Julian Malicki

**Affiliations:** 1 Electroradiology Department, Poznan University of Medical Sciences, 61-866 Poznan, Poland; agnieszka.skrobala@wco.pl (A.S.); adam.ryczkowski@wco.pl (A.R.); wiktoria.suchorska@wco.pl (W.S.); igor.piotrowski@wco.pl (I.P.); natalia.matuszak@wco.pl (N.M.); julian.malicki@wco.pl (J.M.); 2 Medical Physics Department, Greater Poland Cancer Centre, 61-866 Poznan, Poland; piotr.romanski@wco.pl (P.R.); dorota.borowicz@wco.pl (D.B.); kinga.graczyk@wco.pl (K.G.); 3 Radiobiology Laboratory, Medical Physics Department, Greater Poland Cancer Centre, 61-866 Poznan, Poland; katarzyna.kulcenty@wco.pl

**Keywords:** out-of-field doses, dose reduction, prostate cancer, phantom

## Abstract

The aim of the study was to determine the influence of a key treatment plan and beam parameters on overall dose distribution and on doses in organs laying in further distance from the target during prostate SBRT. Multiple representative treatment plans (n = 12) for TrueBeam and CyberKnife were prepared and evaluated. Nontarget doses were measured with anionization chamber, in a quasi-humanoid phantom at four sites corresponding to the intestines, right lung, thyroid, and head. The following parameters were modified: radiotherapy technique, presence or not of a flattening filter, degree of modulation, and use or not of jaw tracking function for TrueBeam and beam orientation set-up, optimization techniques, and number of MUs for CyberKnife. After usual optimization doses in intestines (near the target) were 0.73% and 0.76%, in head (farthest from target) 0.05% and 0.19% for TrueBeam and CyberKnife, respectively. For TrueBeam the highest peripheral (head, thyroid, lung) doses occurred for the VMAT with the flattening filter while the lowest for 3DCRT. For CyberKnife the highest doses were for gantry with caudal direction beams blocked (gantry close to OARs) while the lowest was the low modulated VOLO optimization technique. The easiest method to reduce peripheral doses was to combine FFF with jaw tracking and reducing monitor units at TrueBeam and to avoid gantry position close to OARs together with reduction of monitor units at CyberKnife, respectively. The presented strategies allowed to significantly reduce out-of-field and nontarget doses during prostate radiotherapy delivered with TrueBeam and CyberKnife. A different approach was required to reduce peripheral doses because of the difference in dose delivery techniques: non-coplanar using CyberKnife and coplanar using TrueBeam, respectively.

## 1. Introduction

The main objective of radiation therapy is to eliminate cancer cells while sparing healthy tissues, particularly the organs at risk (OAR), which may be located near or distant from the target region. Although all modern radiotherapy techniques are capable of precisely delivering the radiation dose to the target, all of these techniques—especially dose-escalated approaches and those composed of tens to hundreds of beams—inevitably deliver radiation outside of the target and of the treatment fields, potentially increasing the risk of developing a secondary tumor [1,2,3,4].

The nontarget radiation doses produced by advanced techniques differ according to the specific parameters required for a given technique and due to model-specific differences among linear accelerators (e.g., gantry design, beam collimating, and shielding systems) [5]. They occur in places laying inside of one or more beams (primary radiation) or are due to scattered radiation only (out-of-field doses). Studies have evaluated nontarget doses in commonly used linacs, including the Clinac2300 (Varian) [6,7], Elekta Synergy (Elekta), and Siemens Primus [8]. However, relatively few studies have compared nontarget doses in more modern linacs such as TrueBeam (Varian) and CyberKnife (Accuray). A few studies have compared some modalities (3DCRT, IMRT, VMAT) to CyberKnife, but our understanding of the effects of non-coplanar techniques on out-of-field doses remains poor [3,9].

Apart from the accelerator model and radiotherapy technique, other parameters can also significantly influence out–of-field doses in prostate cancer (PCa) radiotherapy, including the beam energy (with or without flattening filter) [10,11], the size and shape of the irradiated field defined by the collimator jaws (jaw tracking function) [12], the presence and arrangement of the multileaf collimator (MLC) [13], the application of beam modifiers, and the position/orientation of these beams (or modifiers) relative to the target volumes. The optimization and degree of modulation with beam intensity [12], type of calculation algorithm, number of monitor units (MUs) [8], and the angles and direction of beam entry (non-coplanar techniques) will depend on the specific technique. The degree of modulation might also affect the nontarget doses. Various studies have assessed nontarget doses produced by different beam energies and techniques [7,13] and other studies have sought to determine the influence of treatment parameters on out-of-field doses [1,8,10]. However, it is still not clear which treatment parameters have the greatest impact on nontarget doses and how to reduce the nontarget dose for a particular technique. The AAPM TG-158 report provides valuable information to assist physicists in designing clinical approaches to measure and calculate nontarget doses and also provides a description of the methods available to reduce these doses [13]. However, reliable comparisons of the available studies are hindered by the influence of the measurement conditions on dose levels [8].

In this context, the aim of this study was to determine the influence of key treatment and beam orientation set-up parameters on the overall dose distribution in a phantom model of prostate cancer and to identify the parameters that have the greatest impact on nontarget doses in selected OARs. All treatment plans were prepared for TrueBeam and CyberKnife linacs.

## 2. Materials and Methods

### 2.1. Phantom for Nontarget Doses

**Figure 1:**
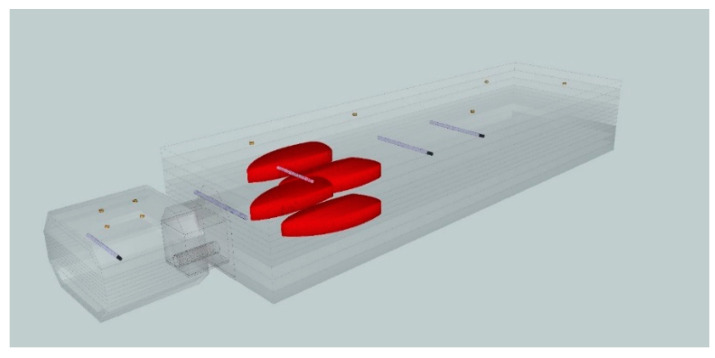
A quasi-anthropomorphic phantom with built-in elements to simulate lung and head to measure out-of-field doses at five selected points representing the prostate gland (+0 cm from the target/central axis), intestine (+15 cm), right lung (+35 cm), thyroid (+54 cm), and head (+75 cm).

To measure nontarget doses we constructed a quasi-anthropomorphic (Q-H) poly(methyl methacrylate) PMMA phantom with built-in elements to simulate lung and bone tissue [14]. Tomographic scans of the assembled Q-H phantom were performed using Siemens SOMATOM Definition AS (120 kV X-ray Tube Voltage, 1 mm slice thickness) according to our onsite protocol. We evaluated five points representing different organs located less than and greater than 20 cm from the central axis (CAX), as follows: prostate (+0 cm from CAX), intestine (+15 cm), right lung (+35 cm), thyroid (+54 cm), and head (+75 cm). All points (except the lung) were located along the same axis at a depth of 11 cm (Figure 1). The lungs were located asymmetrically (6 cm from the long axis). The phantom included built-in gold markers for precise positioning and image-guided radiotherapy (IGRT).

### 2.2. Treatment Plans and Accelerators

All treatment plans were designed in a head-first supine position utilizing the SBRT for PCa. The conventional clinical target volume (pseudo-CTV) and OARs were delineated. A margin of 5 mm in all directions was added to the pseudo-CTV to create the planning target volume (pseudo-PTV). The prescription dose was 10 Gy/fraction, covering ≥ 95% of the PTV (maximum dose: <120%).

Multiple treatment plans were created for the CyberKnife, VSI v.11.1 (Accuray Inc., Sunnyvale, CA, USA) and TrueBeam, v. 2.7 (Varian, Medical Systems Inc., Palo Alto, CA, USA) linacs. Plans were created with photon beams (nominal energy: 6 MV) as a standard treatment (considering only the volumes close to the target) and re-computed for each study parameter to reduce out-of-field low dose radiation. All plans met all constraints and clinical criteria, including PTV coverage, dose reduction to surrounding normal tissues, and treatment time. The most representative plans were selected (plans 1–12) for the study (Table 1).

For the TrueBeam, seven plans (1–7) were created using Eclipse software v.15.6 (Varian, Medical Systems Inc., Palo Alto, CA, USA). The maximum dose rate was 600 MU/min (6 MV FF) and 1400 MU/min (6 MV FFF). The following parameters were modified to determine their impact on nontarget radiation dose reductions: radiotherapy technique (3DCRT, IMRT, and VMAT), presence or not of a flattening filter (6 MV FF and 6 MV FFF), degree of modulation/number of MUs, and use or not of jaw tracking function. Five VMAT plans were created: four FFF (plans 1–4) and one FF (plan 5). Three optimization strategies were used to define the PTV to obtain three levels of MUs: standard/high (plan 1), intermediate (30% reduction in MUs, plan 3), and low (50% reduction, plan 2). A 4th plan (plan 4) was created for the high number of MUs using jaw tracking for dose optimization.

Five different CyberKnife treatments plans (plans 8–12) were created using Precision v. 2.0.1.0 software (Accuray Inc., Sunnyvale, CA, USA). The maximum dose rate was 1000 MU/min using the Iris variable aperture collimator with two different aperture sizes (35.0 and 50.0 mm). The following parameters were evaluated to determine their influence on nontarget doses: beam orientation set-up, optimization techniques, and degree of modulation.

Due to the significant effect of the beam orientation set-up (non-coplanar technique) in CyberKnife techniques, the influence of the beam entry direction on doses outside the field was checked, including one field preliminary test (simple geometry) with the gantry positioned at different beam angles (0°, 45° [cranial direction], and 315° [caudal]).

We applied three sequential optimization plans (plans 8–10) with different beam arrangements for CyberKnife: a standard plan with fewer beams in both caudal and cranial directions (plan 8) and two other plans intended to reduce out-of-field doses, one with no caudal beams (plan 9) and another with no cranial beams (plan 10).

To compare the influence of the optimization techniques on nontarget and out-of-field doses, dose optimization was performed with a sequential optimization technique (plan 8) and the VOLO optimizer (plan 11–12). For VOLO optimization, two plans were created, both with fewer beams from the cranial and caudal directions: VOLO standard (plan 11) and VOLO with fewer MUs (plan 12).

All plans were required to pass the dose difference (DD) and distance-to-agreement (DTA) gamma criteria and threshold (3%/2 mm, TH10%) as recommended in TG AAPM 218 [8]. To determine differences between individual SBRT plans, we performed additional analyses using different DD/DTA gamma criteria (L2%/2 mm, and TH10%). The distribution was registered by using 2D-arrays of SRS1000 and 1500 detectors placed in the phantoms: Octavius II and Octavius 4D (PTW Freiburg, Germany). All distributions were analyzed using the 2D or 3D gamma analysis in Verisoft 8.0.1 software (PTW, Freiburg, Germany).

### 2.3. Measurements of Nontarget and Out-of-Field Dose

The ionization chamber was used to measure doses at the five selected points for the 12 different treatment plans (plans 1–12) and for simple geometry (13). For the standard CyberKnife treatment plan (plan 8), doses were measured at four points outside the irradiated volume for 159 individual radiation beams. The influence of specific geometric parameters on the nontarget dose were determined for each gantry position and beam entry direction (left side [L], right side [R], middle [CH], cranial [I], caudal [S], center [CV]). For the standard CyberKnife plan (plan 8), each M (defined as dose without energy correction) was divided by the MU values to enable equivalent comparisons (not dependent on the number of MUs).

Measuring nontarget doses and choosing the appropriate detector is a challenge. Film detectors or thermoluminescent (TLD) detectors are often used due to small sizes and feasibility for measurements in anthropomorphic phantoms. Such passive detectors need much longer time of processing after exposure in comparison to active detectors, which makes their use for study of impact of individual beam parameters on doses impractical. All measurements made with the ionization chambers (repeated three times) were performed under identical conditions (i.e., established dosimeter settings and phantom set-up parameters) to objectively compare the nontarget doses between specific treatment plans at individual measuring points to detect even slight differences between techniques. The Semiflex chamber has a low atomic number Z (<10) and detectors with Z < 13 exhibit a nearly flat energy response, typically by less than 5% to effective energies of 40 keV (compared to 60 Co) [13,15]. For specific out-of-field points, the differences in energy between 6FF and 6FFF photons is small [10], and the dose rate changes as a function of increasing distance. However, since the most important factor is proximity to the radiation field [7,13], no correction factor is needed for relative comparisons. All statistical analyses and graphs were made with the R software program.

## 3. Results

As the distance from the target volume increased, doses outside the target initially decreased rapidly for all techniques, as evidenced by the steep gradient on the dose distribution curves, which had a nearly exponential shape according to the distance from the CAX. Depending on the physics parameters applied and technique used, the distribution shape and dose level changed in specific areas. At the point representing the intestines (located < 20 cm from the CAX), the dose range was 0.59–0.74% of the CAX for the TrueBeam techniques and 0.64–0.90% for CyberKnife. In more distant areas (>20 cm), the range was greater for both techniques: 0.03–0.11% for TrueBeam and 0.12–0.19% for CyberKnife.

### 3.1. Nontarget Doses: TrueBeam

The lowest value (0.59%) for intestines was observed for 3DCRT (plan 7), followed by IMRT (plan 6; 0.63%) and VMAT with FF (plan 5; 0.66%). For the FFF VMAT techniques, dose levels were higher, as follows: 0.73% and 0.74% for the standard (plan 1) and intermediate (plan 3) beam modulation, respectively. Importantly, plans with low beam modulation (plan 2) or jaw tracking (plan 4) for standard modulation resulted in lower doses (0.68% of the CAX dose, representing close to a 7% dose reduction compared to the standard plan).

**Figure 2:**
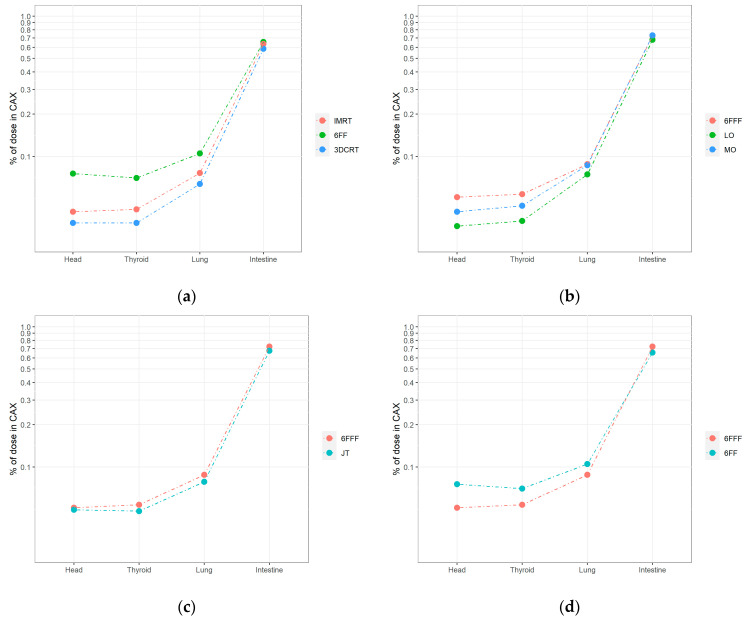

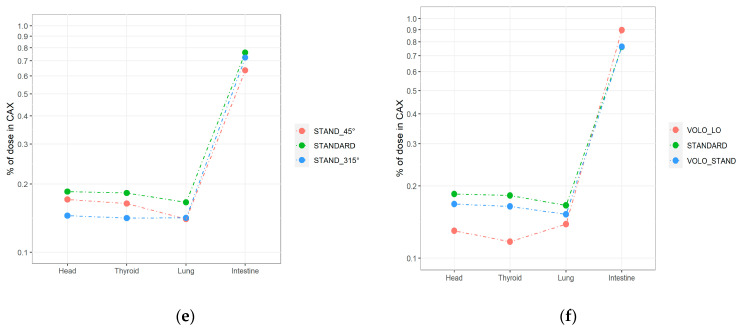
A semilogarithmic comparison of nontarget doses measured by ionization chambers located in the selected points (intestine, right lung, thyroid, and head) under the following conditions for TrueBeam: (**a**) 3DCRT vs. IMRT vs. VMAT (6 MV FF); (**b**) jaw tracking (JT) vs. standard irradiation (6 MV FFF); (**c**) different levels of modulation in VMAT 6 MV FFF plans; (**d**) flattened vs. unflattened beam. The parameters for CyberKnife were: (**e**). limited entry of beams from cranial (45°) and caudal (315°) directions; (**f**) different levels of modulation (LOW vs. HIGH) and algorithms (SEQUENTIAL vs. VOLO).

Dose levels for TrueBeam at points > 20 cm from the CAX decreased more slowly than those located < 20 cm. The highest values were observed for the FF VMAT plan (plan 5) in the lung (0.11%), thyroid (0.07%) and head (0.08%). For the head and thyroid, similar values were obtained for the VMAT plans with the FFF beam (plan 1) with standard modulation (0.05%), jaw tracking (plan 4; 0.05%), and with intermediate beam modulation (plan 3, 0.04%). For the lung, the values were as follows: 0.09% for the standard (plan 1) and intermediate (plan 3) modulation plans. For the plan with jaw tracking (plan 4), the value was 0.08%, which was slightly lower than the standard plan (plan 1). The lowest distant doses were observed for the 3DCRT technique (plan 7: lung, 0.06%; thyroid and head, 0.03%). Similar results were obtained for the VMAT plan using the FFF beam with low beam modulation (plan 2: lung, 0.07% and thyroid and head, 0.03%). A 50% reduction in MUs resulted in an out-of-field (>20 cm) dose reduction of up to 40%, while a 30% reduction in MUs resulted in 20% lower doses compared to the standard high modulation plan. The impact of the key treatment plan parameters and techniques used are shown in Figure 2 as the % CAX dose.

### 3.2. Nontarget Doses for CyberKnife

For simple geometry (plan 13), the highest relative nontarget doses to organs located > 20 cm from the CAX were as follows: 0.19% (head), 0.16% (thyroid), 0.13% (lung) of the dose in CAX for the 45° cranial side beam (gantry head directly above the measurement points). When the gantry head was positioned at 315° caudally of the beam entrance above the legs, doses to the thyroid (0.03%) and head (0.02%) were 5 and 10 times lower than for 45°, respectively. The % CAX dose with the gantry head positioned at 0° was 0.06% for all measurement points > 20 cm. For the intestine, the best distribution was obtained with a beam angle of 0° (0.24% of CAX dose) vs. 45° (0.40%) and 315° (0.81%), with the primary beam located closer to the intestines.

**Figure 3:**
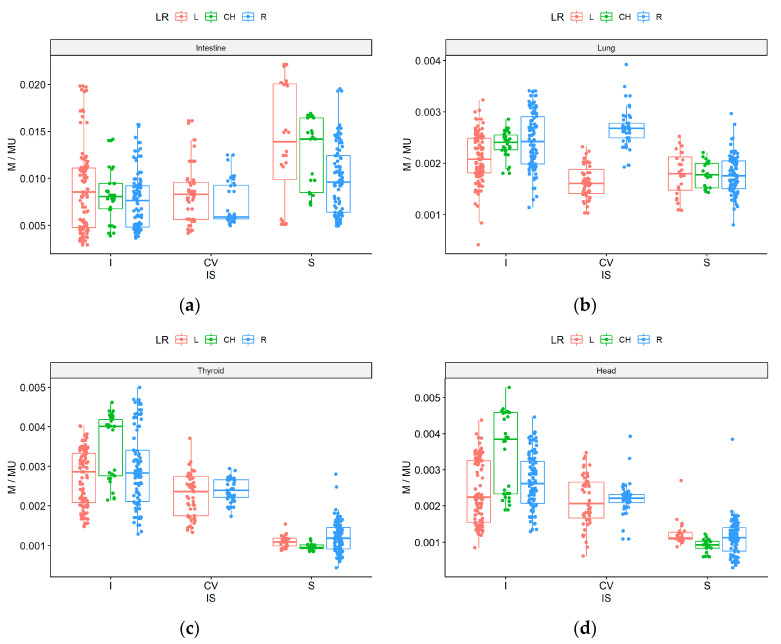
M/MU parameter for CyberKnife standard plan (8) for each of the 159 beams measured outside the target volume according to beam direction: left side (red), right side (blue), middle (green), and cranial (I), caudal (S), center (CV) for intestine (**a**), right lung (**b**), thyroid (**c**), and head (**d**).

The results for each of the 159 beams (given as M/MU) are presented in Figure 3. The influence of the gantry head position on nontarget low doses observed in treatment plan 8, was similar to that observed for simple geometry (plan 13). For the head and thyroid, the lowest values were obtained from the caudal direction side (S), with a median M/MU ratio of 0.0010 mGy/MU. The highest value for the cranial direction was obtained for beams located in the middle (CH), for the head and thyroid, respectively: 0.0038 and 0.0040 mGy/MU, compared to the left/right side (0.0023 and 0.0028 mGy/MU). For the right lung, the lowest doses were obtained on the central (CV) and left position of the gantry head (median: 0.0016 mGy/MU) and the highest values on the central (CV) and right position (0.0027 mGy/MU). A trend towards higher doses was observed when the gantry head was positioned on the right side (vs. the left side) due to the asymmetric localization of the measurement point for the right lung. Note that this trend was not evidenced with caudal beams.

For the intestines, the lowest dose was achieved with beams from the center beam direction and from the right side (median 0.0059 mGy/MU) because the primary beam does not directly reach this measurement point and the gantry head is located far from the intestines. The opposite result was observed for the caudal (S) direction, where the median doses were 0.0140 mGy/MU for the left and CH beam directions. Left side values tended to be higher with a wider spread than those on the right side, probably because the linac is located on the right side of the treatment couch.

**Figure 4:**
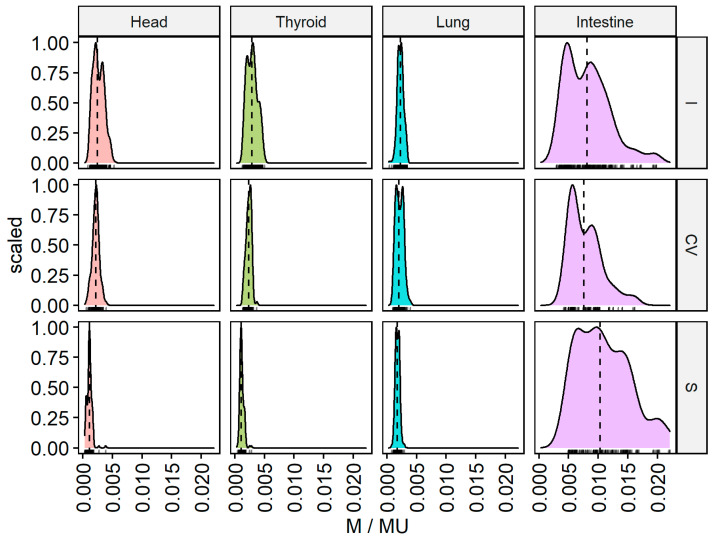
M/MU parameter for CyberKnife standard plan (8) for each of the 159 beams measured outside the irradiated volume for different beam directions: cranial (I), caudal (S), and center (CV) for the intestines, right lung, thyroid, and head.

These measurements are presented as function densities and frequency histograms for a given measurement value (M/MU), as shown in Figure 4. For the intestine, the cranial (I) and caudal (S) beams form the flattest distribution curves (wide dose range but small frequency range). Areas located far from the target volume show narrow individual peaks for the head and thyroid from the caudal direction (S), indicating low dose levels in a narrow range, in contrast to the direction of entry of the beam from the cranial directions (I). The double peaks in the histograms may be attributable to scattered doses from the linac head. Double peaks are also visible for the right lung for the central position of the gantry (CV). By measuring each individual beam, it is possible to empirically differentiate the effect of low doses scattered within the phantom itself from low doses attributable to gantry head scattering.

At distances < 20 cm (i.e., the intestine), the doses ranged from 0.64 to 0.90% of the CAX dose. The lowest values were achieved for the standard treatment plan prepared with blocked beams from the caudal direction (plan 9, 0.64%). The highest value was obtained for the low modulated VOLO optimization (plan 12, 0.90%).

For the lung, the % CAX dose values for plans designed to reduce nontarget doses were similar (0.14–0.15%). For the head and thyroid, plan 9 (blockage of caudal beams) and plan 11 (VOLO optimization) yielded similar values, 0.17% and 0.16%, respectively. The lowest values for the head and thyroid regions were achieved by blocking cranial beams (plan 10), resulting in 0.14% and 0.14%, respectively; and for the low beam modulation VOLO plan (plan 12; 0.12% and 0.13%, respectively). A 20% reduction in MUs resulted in an out-of-field (>20 cm) dose reduction of up to 23% compared to the high modulation plan.

### 3.3. Summary of Results for TrueBeam and CyberKnife

Table 2 shows the nontarget doses obtained with the different radiotherapy techniques.

For the intestine, the highest value (% CAX dose) was obtained with the low beam modulation VOLO plan (plan 12; 0.90%), followed by high and intermediate modulated FFF plans (plans 1 and 3, respectively) and most CyberKnife plans (plans 8, 10–11). For the remaining plans, the % doses were <0.68%, with the lowest value for 3DCRT (0.59%).

Table 2 shows the percentage doses for the various plans. The lowest doses for all analyzed points were obtained with 3DCRT. For low modulation VMAT (plan 2), the nontarget % dose was comparable to 3DCRT but only for distant points. The highest dose percentages were observed for CyberKnife, especially the standard (plans 8–9) and VOLO plans (#11). By contrast, the lowest doses (0.12–0.14%) were obtained for low modulation plans and the plan without caudal beams (0.14%). For CyberKnife, doses > 20 cm from the CAX were 0.1% higher than for TrueBeam, mainly due to the non-coplanar technique (radiation from the primary beam or head leakage) and higher number of MUs (7500–8000 vs. 2500–4800).

## 4. Discussion

This study was performed to determine the impact of modifying different treatment parameters on nontarget dosimetric values, particularly those parameters most relevant in prostate SBRT (CyberKnife, 6FFF, and jaw tracking) as well as 3DCRT and IMRT. We also tested certain parameters (e.g., optimization techniques for CyberKnife and jaw tracking) whose influence on nontarget doses has received little attention to date. The findings presented here extend our knowledge on nontarget low doses, showing the parameters that yield the greatest reduction in out-of-field doses without negatively impacting the quality of the treatment plan.

In general, out-of-field doses are caused by the construction and design of the gantry head (due to scattering from the collimator and leakage through the gantry). However, modern dose escalated, and particularly non-coplanar techniques produce nontarget doses due to the multiple beams reaching the target from variety of directions and modulations [1]. Evaluation of non-target and particularly out-of-field doses is difficult because these doses are greatly dependent on factors related to accelerator construction and to the specific treatment technique [9]. For this reason, as technology continues to advance, it is essential that we evaluate how the new technology and treatment modalities affect low doses outside the target. In this regard, the present study is—to our knowledge—the first to perform complex comparisons for nontarget doses for TrueBeam and CyberKnife.

Some studies include Monte Carlo simulations for simple geometries calculated with high accuracy [16,17] and comparisons of complex techniques or different linacs [9,18], which have provided an overview of low doses produced by different modalities and techniques (e.g., 3DCRT vs. IMRT [1,2]; IMRT vs. Tomotherapy [3]). Other studies have evaluated how changes in a single parameter affects low doses, including MLC [19], field size [20], modulation [13], and FF vs. FFF [10,11]. Despite the value of these studies, simple geometries do not provide an accurate picture of the true clinical situation, and comparisons of different modalities are influenced by many variables.

In an effort to standardize the measurement conditions, we used a purpose-built phantom designed specifically to measure low doses from scattered radiation. We selected the parameters most likely to affect nontarget doses in order to determine how modification of those parameters would influence doses under identical measurement conditions. In this regard, it is important to determine which technical parameters can be adjusted without resulting in a treatment plan that is unsuitable for clinical use, as well as to determine the parameters that cannot be modified. In this regard, ours is the first study to develop a list of modifiable and unmodifiable parameters, thus providing new, valuable information.

### 4.1. TrueBeam

For beams with a flattening filter, the highest doses for all measurement points outside the irradiated volume were obtained with VMAT (vs. IMRT and 3DCRT). Previous studies have shown that 3DCRT results in the lowest out-of-field doses, which is consistent with our findings [2]. For most measured points, low doses were higher for VMAT than for IMRT [1,21], but this was strongly dependent on the degree of modulation and number of MUs [13,17], and the energy used (prostate IMRT is often 15 MV) [22]. It is worth noting that we compared techniques for the same energy (6 MV), although this is not the most common clinical dose.

In regard to the results for FF and FFF beams, out-of-field doses were lower with FFF beams, a finding that is in line with previous reports [10,11]. However, beams with FFF resulted in a higher CAX dose (0.73% vs. 0.66%) in the intestinal region, perhaps due to the influence of the dominant radiation source at larger distances (>20 cm) from the target, where collimator scattering and gantry leakage play an important role, whereas scatter from the target volume is the main cause of out-of-field doses at closer distances [13]. Studies have shown that SBRT results in lower out-of-field doses (regardless of distance from the CAX) when no flattening filter is used [10].

The out-of-field dose can be decreased by reducing the number of MUs. For example, we found that decreasing MUs by 50% resulted in an out-of-field (>20 cm) dose reduction of 40% while a 30% decrease in MUs lowered the dose by 20% compared to the standard high modulation plan. This effect is visible in the areas most distant from the field (thyroid and head), where more MUs increase the dominant effect (collimator scattering). Interestingly, no major differences were found between high and intermediate modulation for the lungs and intestines. The low degree of modulation for FF beams reduces out-of-field doses in a similar manner to that observed with 3DCRT with FF. High modulation, which changes treatment plan conformality, influences nontarget doses due to higher scatter from dynamic MLC and a generally higher number of MUs. Near the field edge, the degree of modulation can significantly impact out-of-field doses. At greater distances, collimator scattering (mainly MLC) and scattering from the accelerator head (head shielding and coplanarity of beam entry) play a larger role. For highly modulated beams, collimator scatter and MUs are higher.

Jaw tracking follows the MLC field shape, thereby reducing leakage and lowering dose to OARs by up to 2% in the V5 and V20 volumes [12]. Reducing the dose around the target and minimizing leakage through the MLC reduces out-of-field doses, with less impact in the thyroid and head.

For TrueBeam, the most effective way to reduce peripheral doses is to use 3DCRT and VMAT techniques without a flattening filter and fewer MUs. However, for 3DCRT treatments, this approach significantly reduces conformality; moreover, when fewer MUs are used, it is essential to verify that dose distribution to the target and OARs is acceptable. VMAT has other advantages, including improved target volume conformity, particularly in volumes with complex concave shapes, and better sparing of healthy tissues and OARs. FFF VMAT with jaw tracking reduces low dose out-of-field radiation without sacrificing the quality of the SBRT treatment plan and may even improve beam modeling accuracy.

### 4.2. CyberKnife

The gantry head position can influence nontarget doses [23,24], as we observed in treatment plan 8, similar to the effects observed for simple geometry (plan 13). Beams with a cranial orientation resulted in higher nontarget doses to organs located >20 cm from the target area. When the gantry head was positioned on the abdominal side and legs (315/S), the doses to these organs were lower than those observed with the gantry head on the cranial side (Figure 3). For the intestines, the best dose distribution was a beam from the center direction and from the right side of the phantom (the linac is located on the right side of the table) because the primary beam does not directly involve the intestine and because the gantry is located far from the intestines. For the right lung, the lowest doses were obtained from the central and left positions of the gantry head. For the head and thyroid, the lowest doses were obtained from the caudal direction and centrally. The dose range from the right side was wider than from the left. The highest doses were obtained when the gantry head was located above these areas (i.e., cranial direction in the center). When MUs were reduced by 20%, distant out-of-field doses decreased by up to 23% compared to high modulation plans.

### 4.3. Methods to Reduce Low Doses

Although a wide range of treatment plans and techniques can be used for prostate radiotherapy, it is important to keep in mind that modifying treatment parameters to reduce nontarget doses >20 cm from the target will increase the doses closer to the irradiated field.

For TrueBeam, the easiest method to reduce out-of-field is to combine FFF with jaw tracking while keeping the number of MUs as low as possible (while ensuring appropriate conformality). For highly selected cases in which avoiding low dose out-of-field radiation is of great importance, 3DCRT can be used. However, similar effects can be achieved with low modulation with better conformality.

For CyberKnife-based treatments, nontarget doses can be limited by blocking the beams to certain risk areas, or by avoiding positioning the gantry over the OAR. When blocking beam entry from the required directions, special attention should be paid to whether such a plan is technically feasible on the accelerator. Pre-verification of the feasibility of treatment plans that have been modified to reduce low dose radiation may achieve lower passing rates; thus, it is essential to ensure that the plan meets all criteria and assumptions and is actually feasible. The simplest solution in prostate cancer would be to irradiate the patient in an inverted position (i.e., in the supine position, feet first) with the most important OARs located far from the gantry head. An interesting option would be to minimize MUs, either by creating less modulated plans, or using optimization algorithms, which allows for the reduction of MUs (e.g., VOLO).

### 4.4. Study Strength and Limitations

Given the difficulty of measuring peripheral doses, which are usually low and possess the important impact of inter-accelerator variability (co-planar vs. non-coplanar), we sought to identify general strategies to reduce low doses, which can vary highly due the multiple factors involved. The doses described here reflect the results of individual treatment plans for a given technique rather than the determination of doses for any specific, commonly-used technique. The use of different treatment plan parameters would likely have led to slightly different effects on peripheral dose distributions. Ultimately, the measurements should consider correction factors to allow for assessment of the absolute dose, as well as the possibility of comparing the doses not only to those occurring in a given location, but also the comparison between another points (e.g., head vs. lung).

In contrast to previous studies, we compared a wide range of different plans and treatment modalities, particularly co-planar and non-coplanar, under the same measurement conditions, which makes this study unique and highly valuable as the first such comprehensive study of low-dose, nontarget radiation in prostate cancer. It is imperative that medical physicists understand the relative magnitude of dose levels outside of the target volume as well as the methods available to manage these doses.

## 5. Conclusions

The presented strategies allowed us to significantly reduce out-of-field and nontarget doses during prostate radiotherapy delivered with TrueBeam and CyberKnife. A different approach was required to reduce peripheral doses because of the difference in dose delivery techniques: non-coplanar at CyberKnife and coplanar using TrueBeam, respectively. The easiest method to reduce peripheral doses was to combine FFF with jaw tracking and reducing monitor units at TrueBeam and to avoid gantry position close to OARs together with reduction of monitor units at CyberKnife, respectively.

## Figures and Tables

**Table 1:** Description of plan design, modulation, energy, and dosimetric parameters.

	Abbreviation/ Plan Name	Machine Algorithms	Techniques	Energy	Modulation	MU	Beam Orientation Set-Up	Dosimetric Verification (3%/2 mm and L2%/2 mm)
1	6FFF	Anisotropic Analytical Algorithm (AAA, v. 15.6); photon optimization algorithm (PO, v. 15.6.05)	VMAT	6FFF	High	4790	gantry rotation angles 181–179 (30°), 179–181 (330°), and 220–140 (330°)	95.1%
2	LO		VMAT	6FFF	Low	2431		99.8%
3	MO		VMAT	6FFF	Middle	3323		97.6%
4	JT		VMAT	6FFF	High with Jaw Tracking	4738		94.7%
5	6FF		VMAT	6FF	High	3296		96.1%
6	IMRT		IMRT	6FF	Low	1790	5 beams with gantry rotation angles: 0°, 100°, 150°, 250°, and 300°	98.6%
7	3DCRT	Anisotropic Analytical Algorithm (AAA, v. 15.6);	3DCRT	6FF	NA	NA	7 beams with gantry rotation angles: 0°, 50°, 100°, 145°, 215°, 250°, and 300°	95.8%
8	CK_STANDARD	Ray-tracing dose calculation algorithm (Precision v. 2.0.1.0) optimization algorithm the sequential optimizer	Non-Coplanar	6FFF	High	7979	159 beams, 40 nodes	100%; 98.5%
9	CK_45°		Non-Coplanar	6FFF	High with Blocked Caudal Beams 45°	7588	161 beams, 40 nodes	99.7%; 94.2%
10	CK_315°		Non-Coplanar	6FFF	High with Blocked Cranial Beams 45°	7502	125 beams, 28 nodes	94.2%; 80.5%
11	VOLO_STAND	Ray-tracing dose calculation algorithm; (Precision v. 2.0.1.0); optimization algorithm the VOLO optimizer	Non-Coplanar	6FFF	High	8217	178 beams, 30 nodes	99.7%; 97.4%
12	VOLO_LO		Non-Coplanar	6FFF	Low	6483	113 beams, 33 nodes	99.8%; 96.0%.
13	SG	NA	Static/Simple Geometry	6FFF	NA	7000	3 beams: 0°, 45° (cranial), 315° (caudal)	NA

Abbreviations: MU, monitor units; VMAT, volumetric modulated arc therapy; IMRT, intensity-modulated radiation therapy; 3DCRT, three-dimensional conformal radiotherapy; FFF, flattening filter free; FF, flattening filter; LO, low degree of modulation; MO, intermediate degree of modulation; JT, jaw tracking; CK, CyberKnife; VOLO, VOLO optimization technique; SG, simple geometry plan; AAA_PO, Anisotropic Analytical Algorithm (AAA, v. 15.6); PO, photon optimization algorithm; PRECISION, Precision v. 2.0.1.0 optimization algorithm; SEQUENTIAL, sequential optimization technique; NA, not applicable.

**Table 2:** Summary of nontarget doses for different radiotherapy and parameters for areas near the field (intestine < 20 cm) and distant from the irradiated volume (lung, thyroid, head) presented as % of CAX dose.

% of Central Axis Dose				
Organ at Risk				
Plan Name	Intestine	Right Lung	Thyroid	Head
VMAT_6FFF	0.73	0.09	0.05	0.05
VMAT_6FFF_LO	0.68	0.07	0.03	0.03
VMAT_6FFF_MO	0.74	0.09	0.04	0.04
VMAT_6FFF_JT	0.68	0.08	0.05	0.05
VMAT_6FF	0.66	0.11	0.07	0.08
IMRT_6FF	0.63	0.08	0.04	0.04
3DCRT_6FF	0.59	0.06	0.03	0.03
STANDARD	0.76	0.17	0.18	0.19
STAND_45°	0.64	0.14	0.16	0.17
STAND_315°	0.72	0.14	0.14	0.14
VOLO_STAND	0.76	0.15	0.16	0.17
VOLO_LO	0.90	0.14	0.12	0.13

## Data Availability

The data that support the findings of this study are available from the corresponding author upon request.
